# Identification of the miRNA-mRNA regulatory pathways and a miR-21-5p based nomogram model in clear cell renal cell carcinoma

**DOI:** 10.7717/peerj.10292

**Published:** 2020-11-04

**Authors:** Yiqiao Zhao, Zijia Tao, Xiaonan Chen

**Affiliations:** Department of Urology, Shengjing Hospital of China Medical University, Shenyang, Liaoning, China

**Keywords:** MicroRNAs, Clear cell renal cell carcinoma, Regulatory network, Bioinformatics methods

## Abstract

**Background:**

The purpose of this study was to determine the key microRNAs (miRNAs) and their regulatory networks in clear cell renal cell carcinoma (ccRCC).

**Methods:**

Five mRNA and three microRNA microarray datasets were downloaded from the Gene Expression Omnibus database and used to screen the differentially expressed miRNAs (DEMs) and differentially expressed genes (DEGs). Gene ontology enrichment analysis and Kyoto Encyclopedia of Genes and Genomes pathway analysis were performed with Metascape. A miRNA-mRNA network was mapped with the Cytoscape tool. The results were validated with data from The Cancer Genome Atlas (TCGA) and qRT-PCR. A nomogram model based on independent prognostic key DEMs, stage and grade was constructed for further investigation.

**Results:**

A total of 26 key DEMs and 307 DEGs were identified. Dysregulation of four key DEMs (miR-21-5p, miR-142-3p, miR-155-5p and miR-342-5p) was identified to correlate with overall survival. The results were validated with TCGA data and qRT-PCR. The nomogram model showed high accuracy in predicting the prognosis of patients with ccRCC.

**Conclusion:**

We identified 26 DEMs that may play vital roles in the regulatory networks of ccRCC. Four miRNAs (miR-21-5p, miR-142-3p, miR-155-5p and miR-342-5p) were considered as potential biomarkers in the prognosis of ccRCC, among which only miR-21-5p was found to be an independent prognostic factor. A nomogram model was then created on the basis of independent factors for better prediction of prognosis for patients with ccRCC. Our results suggest a need for further experimental validation studies.

## Introduction

Renal cell carcinoma is one of the most fatal cancers in the genitourinary system in adults (Siegel, Miller & Jemal, 2018). Its most common subtype is clear cell renal cell carcinoma (ccRCC) (Cheville et al., 2003). Owing to this malignancy’s recurrence and resistance to chemotherapy, the mortality among patients with ccRCC remains high(Lane & Kattan, 2008; Rini, Campbell & Escudier, 2009). Therefore, exploration of ccRCC therapies at the molecular level is urgently needed.

MicroRNAs (miRNAs) are a recently discovered class of small noncoding RNAs (Tran & Hutvagner, 2013), most of which are initially transcribed by RNA polymerase II as long primary transcripts characterized by a hairpin structure. These pre-miRNAs contain stem-loop structures, a 5′-end cap and a 3′-poly (A) tail (Saj & Lai, 2011), which have been found to play important roles in various biological functions such as proliferation, differentiation and apoptosis (Calin & Croce, 2006). miRNAs regulate target gene expression at the post-transcriptional level in various cancers, such as thyroid cancer (Tang et al., 2018) and breast cancer (Li et al., 2017).

In recent years, researchers have reported associations between miRNAs and ccRCC pathogenesis (Braga et al., 2019). For example, miR-381 has been identified as a potential biomarker that suppresses ccRCC cell metastasis and cell proliferation (Chan et al., 2019), and the combination of miR-141 and miR-155 has been found to be able to discriminate ccRCC samples from benign samples (Jung et al., 2009). Qi et al. (2019) revealed that different stages of ccRCC have distinct miRNA profiles. Furthermore, a three-miRNA signature was calculated by Luo et al. (2019) as a prognostic biomarker for patients with ccRCC.

**Figure 1:**
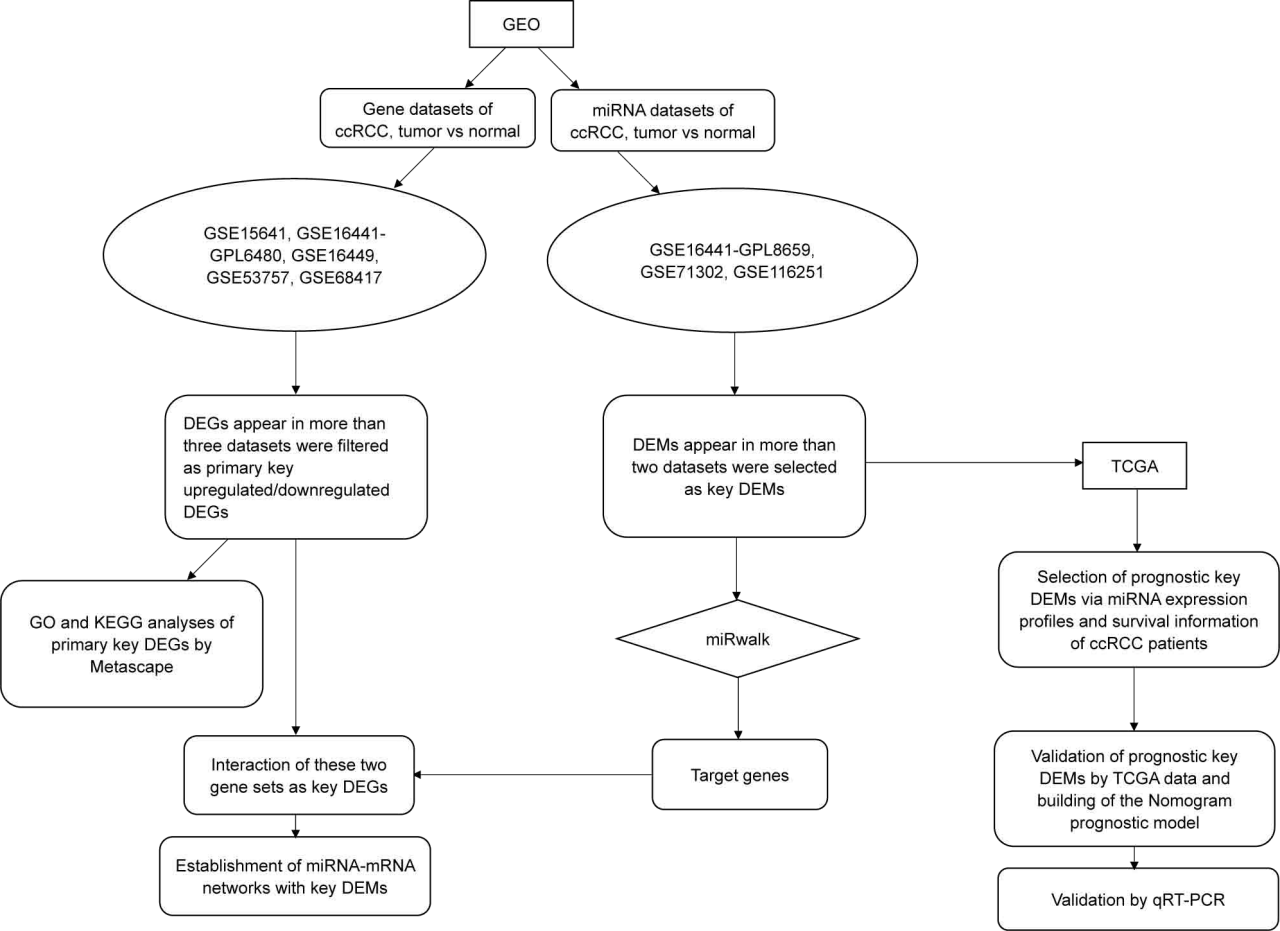
The flow chart of this study. Except for the steps using online tools (Metascape and miRwalk, which were mentioned in the figure), most procedures such as data mining, statistical analysis and validation were achieved by R software (version 3.5.1).

In this study, we downloaded miRNA and gene datasets (three and five, respectively) and used bioinformatics methods to select differentially expressed genes (DEGs) and differentially expressed miRNAs (DEMs), which were used for functional and pathway analyses. We initially performed validation by using data from The Cancer Genome Atlas (TCGA) and quantitative real-time PCR (qRT-PCR), and we initially performed overall survival (OS) analysis with TCGA clinical data. To improve prediction, we then established a nomogram model consisting of has-miR-21-5p, stage and grade. The overall workflow of our study is illustrated in Fig. 1.

## Methods

### Gathering of relevant microarray data

The microarray expression profiling data of miRNAs (GSE16441–GPL8659, GSE71302 and GSE116251) and genes (GSE15641, GSE16441–GPL6480, GSE16449, GSE53757 and GSE68417) were obtained from Gene Expression Omnibus (GEO; http://www.ncbi.nlm.nih.gov/geo/). The details of the microarray datasets are provided in Table 1.

**Table 1:** Basic information of GEO datasets used in this study. In this table, we integrated the basic information of GEO datasets which we used as primary analysis, including their Accesion ID, the PMID of researches including these datasets, the platform of these datasets, number of tumor samples and healthy control samples, and their experiment type.

Accession/ID	PMID	Platform	Number of ccRCC tissues	Number of normal tissues	Gene/MicroRNA
GSE15641	16115910	GPL96	32	23	Gene
GSE16441	20420713, 26941587	GPL6480	17	17	Gene
GSE16449	22363672	GPL6480	52	18	Gene
GSE53757	24962026	GPL570	72	72	Gene
GSE68417	26670202	GPL6244	29	14	Gene
GSE16441	20240713	GPL8659	17	17	MicroRNA
GSE71302	26248649	GPL10850	5	5	MicroRNA
GSE116251	30201497	GPL25243	18	18	MicroRNA

**Notes.**

Abbreviations GEO gene expression Omnibus ccRCC Clear Cell Renal Cell Carcinoma PMID PubMed ID

### Data preprocessing and DEG/DEM analysis

Within each GEO dataset, using the “Limma” package for R v3.5.1, we selected miRNAs and genes with |log fold change (FC) |>1 and adjusted *p*-value of <0.05 as DEMs and DEGs. Three Venn diagrams were constructed with the online Venn diagram drawing tool Draw Venn Diagram (http://bioinformatics.psb.ugent.be/webtools/Venn/). Genes found in interactions in at least three gene datasets were considered the primary key DEGs, whereas miRNAs appearing in more than two miRNA datasets were considered the key DEMs.

### Enrichment analysis of DEGs

Gene ontology (GO) analysis and Kyoto Encyclopedia of Genes and Genomes (KEGG) pathway analysis were performed in Metascape (Zhou et al., 2019) (http://metascape.org/gp/index.html#/main/step1). Key DEGs were uploaded to Metascape, and related GO and KEGG pathway enrichment analyses were performed. The cut-off criterion was set at a *p*-value of <0.05, a minimum count of 3 and an enrichment factor (the ratio between the observed counts and the counts expected by chance) >1.5.

### Construction of the miRNA-target gene regulatory network

To determine target genes of the key DEMs and the interactions among them, we downloaded data from miRWalk (Dweep et al., 2011) (version 3.0; http://mirwalk.umm.uni-heidelberg.de/). For each miRNA, key DEM–target gene intersection data were downloaded with standards of “min-p-value” = 1; “position” = 3UTR, 5UTR and CDS, and three databases (TargetScan, miRDB and miRTarBase) were used. Those target genes were regarded as candidate genes. The interactions between the candidate genes and the primary key DEGs were denoted key DEGs within the list; genes targeted by at least three miRNAs were screened as target nodes, whereas the corresponding miRNAs were considered source nodes. Cytoscape 3.6.1 was used for visualization of the miRNA–mRNA regulatory network.

### Further validation by TCGA data

On the basis of the expression data and clinical data from the “TCGA-KIRC” dataset, we constructed Kaplan–Meier plots with the “survival” package in R v3.5.1. We further selected key DEMs that could be considered independent prognostic factors via univariate and multivariate Cox regression analyses. We then combined the independent prognostic miRNAs with stage and grade to generate a model for better prediction of patient prognosis. Moreover, two-tailed t-tests were performed on the expression levels of these prognostic miRNAs to validate whether our results were reasonable. Box-plots were constructed in GraphPad Prism 7.0.4 for visualization.

### Validation by qRT-PCR from clinical specimens of ccRCC

Further validation was performed via qRT-PCR of the four prognostic key DEMs in 15 pairs of tumor and matched normal tissues (*n* = 30; the clinical and pathologic characteristics of these 15 patients are listed in Table S1.

Detailed procedures of this step are described in our previous article (Chen et al., 2016). The primers for PCR are shown in Table S2. U6 was used as an internal control to normalize the results. Threshold cycle (Ct) values were calculated after each PCR reaction. Each sample was tested in triplicate, and the relative quantification equation (RQ = 2^−ΔΔCt^) was used for evaluating relative miRNA expression. The expression data of all 15 pairs of ccRCC samples and matched normal samples are listed in Table S3. This study was approved by Shengjing Hospital Ethics Committee (2017PS012J).

## Results

### Screening of key DEMs and primary key DEGs according to interactions among GEO datasets

**Figure 2:**
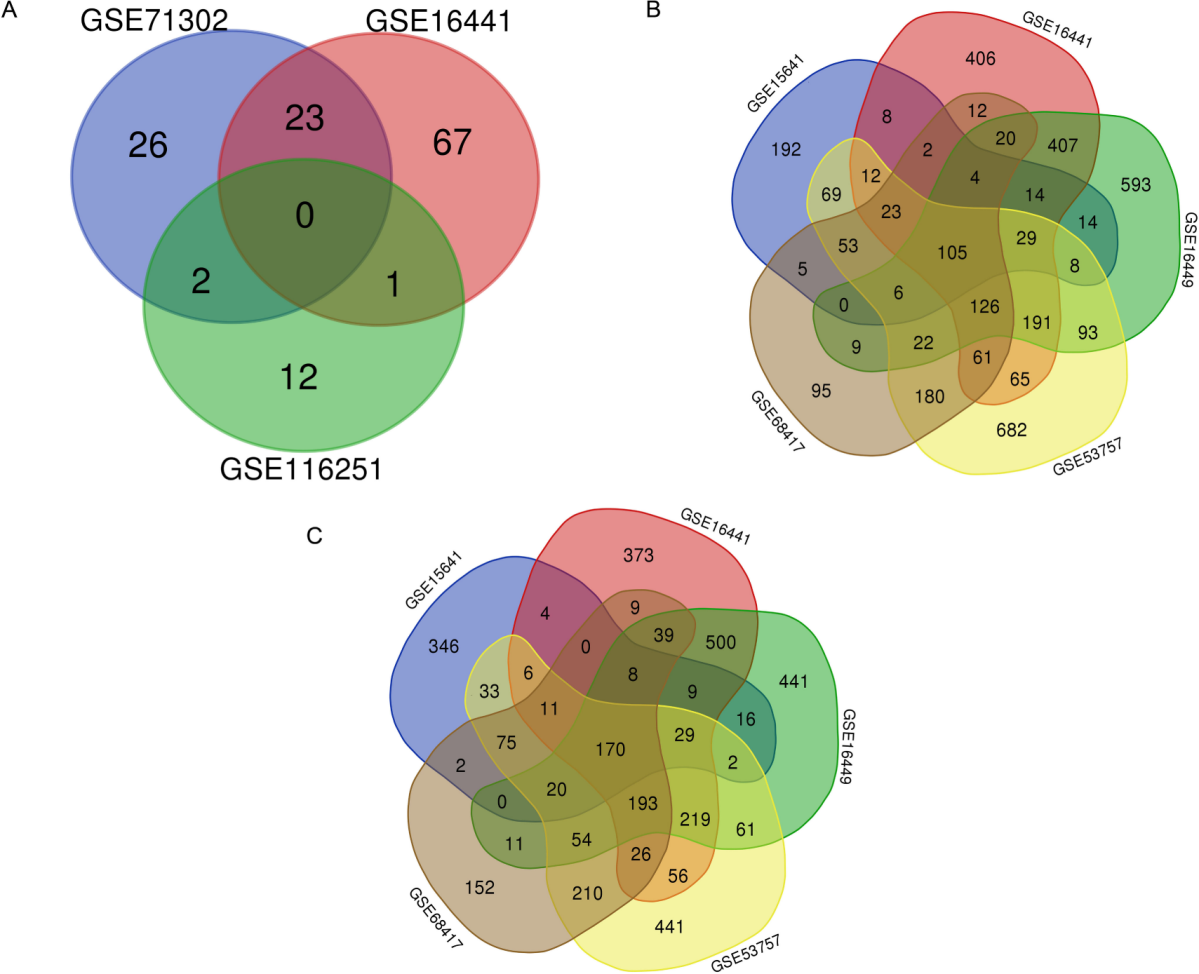
Selection of key DEMs and primary DEGs. (A) Selection of key DEMs through the interaction of more than two datasets; (B) Filteration of primary key upregulated DEGs of by inclusion of DEGs appeard in more than three datasets. (C) Genes which showed up in more than three datasets were regarded as primary key downregulated DEGs.

A total of 7,022 DEGs and 131 DEMs (Fig. 2A) were identified. Within these DEMs, miRNAs that appeared in more than two datasets, such as miR-21, miR-142-3p, miR-155-5p and miR-342-5p, were filtered and considered 26 key DEMs (Supplementary file 1). After the miRWalk 3.0 procedure, 3903 candidate genes were obtained. These candidate genes were found to interact with 3506 upregulated DEGs (Fig. 2B) and 3516 downregulated DEGs (Fig. 2C). A total of 167 key upregulated and 140 key downregulated DEGs were obtained.

**Figure 3:**
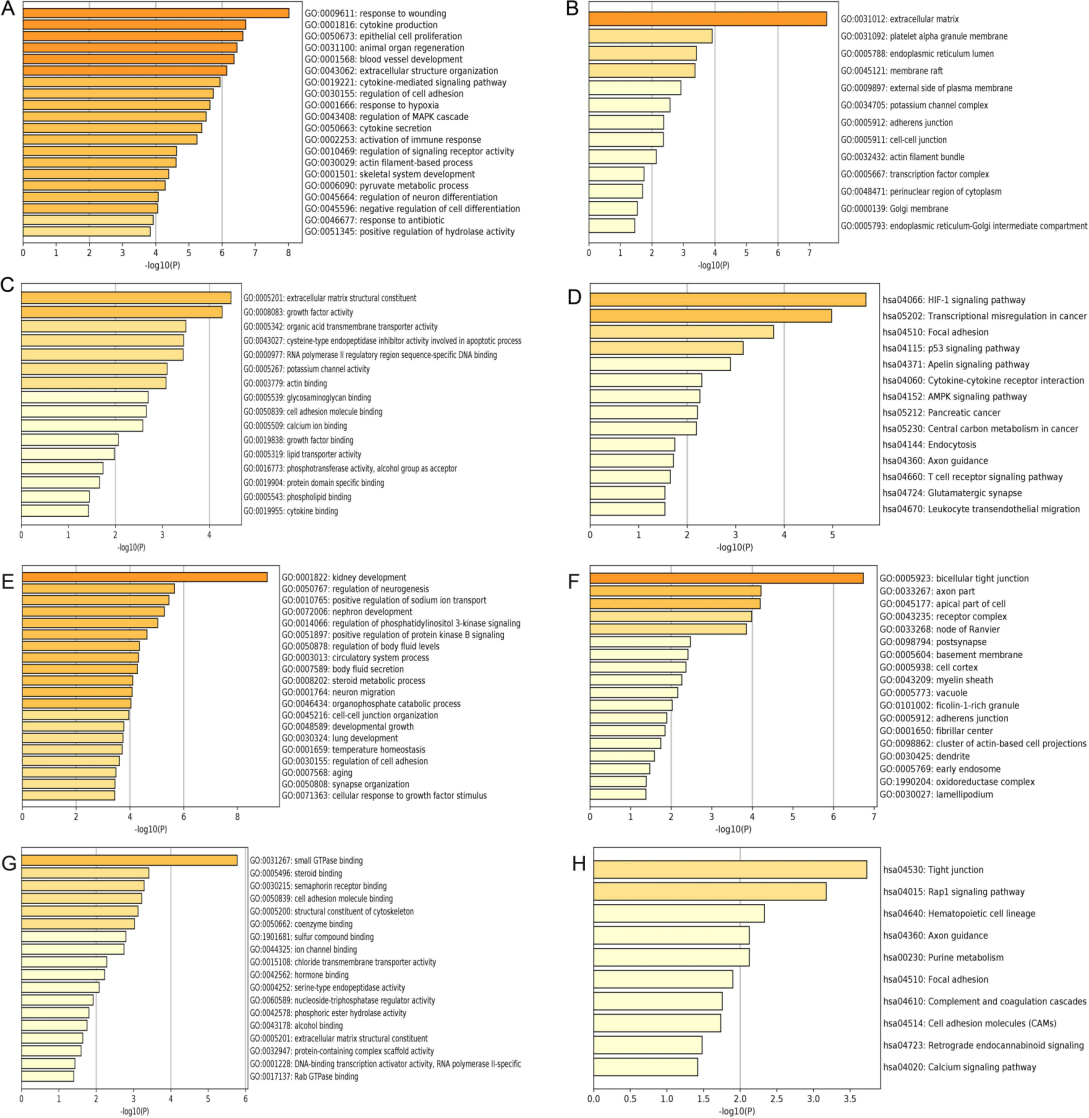
GO and KEGG pathway enrichment analysis of primary key DEGs. (A & E) The results of GO biological process analysis of upregulated DEGs and downregulated DEGs. (B & F) The results of GO cellular component analysis of upregulated DEGs and downregulated DEGs. (C & G) The results of GO molecular function analysis of upregulated DEGs and downregulated DEGs. (D & H) The results of KEGG pathway enrichment of upregulated DEGs and downregulated DEGs.

**Figure 4:**
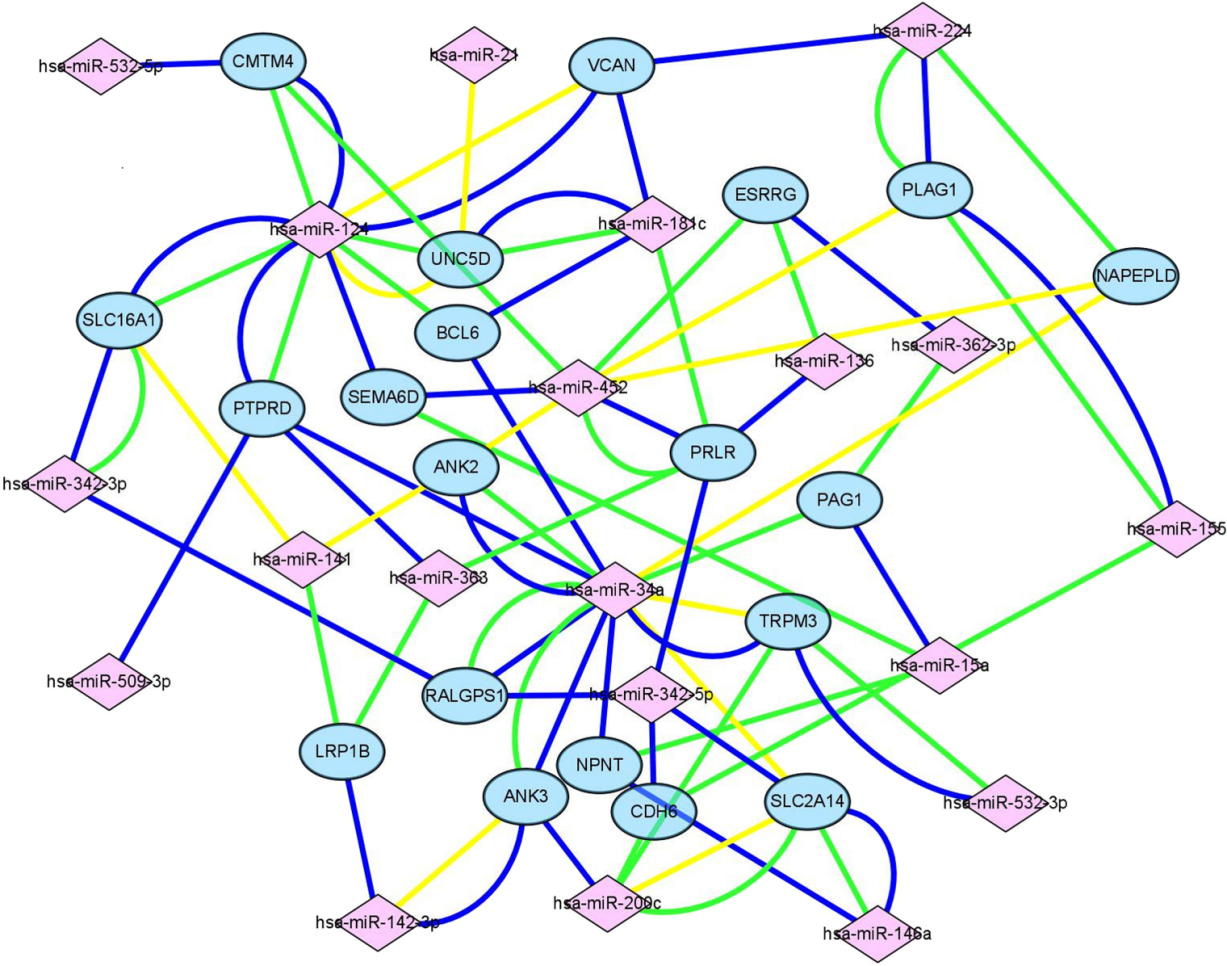
Construction of mIRNA-mRNA regulatory network. Construction of regulatory network consists of miRNAs and mRNAs. Each mRNA was targeted by at least three miRNAs, in this figure, pink square nodes represent miRNA while mRNAs were showen as blue nodes. Lines in different color meant the results of different databases (yellow line, miRTarbase; green line, TargetScan; blue line, miRDB).

### GO and KEGG enrichment analyses of primary key DEGs

GO analysis results showed that the up-regulated DEGs were significantly enriched in biological processes including response to wounding, cytokine production, epithelial cell proliferation, animal organ regeneration and blood vessel development (Fig. 3A). Downregulated DEGs were significantly enriched in biological processes including kidney development, regulation of neurogenesis, positive regulation of sodium ion transport, nephron development and regulation of phosphatidylinositol 3-kinase signaling (Fig. 3E). For cell components, upregulated DEGs were significantly enriched in extracellular matrix, platelet alpha granule membrane and endoplasmic reticulum lumen (Fig. 3B). Downregulated DEGs were significantly enriched in bicellular tight junction, axon part, apical part of cell and receptor complex (Fig. 3F). Moreover, 16 GO molecular function were over-represented in the upregulated DEGs, including extracellular matrix structural constituent, growth factor activity, organic acid transmembrane transporter activity, cysteine-type endopeptidase inhibitor activity involved in apoptotic process, and RNA polymerase II regulatory region sequence-specific DNA binding (Fig. 3C), whereas the downregulated DEGs were significantly enriched in 18 GO molecular functions, including small GTPase binding, steroid binding, semaphorin receptor binding cell adhesion molecule binding and structural constituent of cytoskeleton (Fig. 3G). KEGG pathway analysis showed that the upregulated DEGs were significantly enriched in the HIF-1 signaling pathway, transcriptional misregulation in cancer and focal adhesion (Fig. 3D). Downregulated DEGs were significantly enriched in tight junction, Rap1 signaling pathway and hematopoietic cell lineage (Fig. 3H).

### Construction of an miRNA-mRNA network of primary key DEGs and key DEMs

Target genes were screened with miRTarBase, miRDB and TargetScan. A total of 20 mRNAs, which were targeted by at least three key DEMs, were selected for the construction of the network. For example, BCL6 had connections with miR-124, miR-181c and miR-34a. The miRNA-mRNA network (Fig. 4) was mapped in Cytoscape 3.6.1.

### Overall survival analysis of key DEMs and construction of a nomogram model on the basis of TCGA data along with qRT-PCR validation

**Figure 5:**
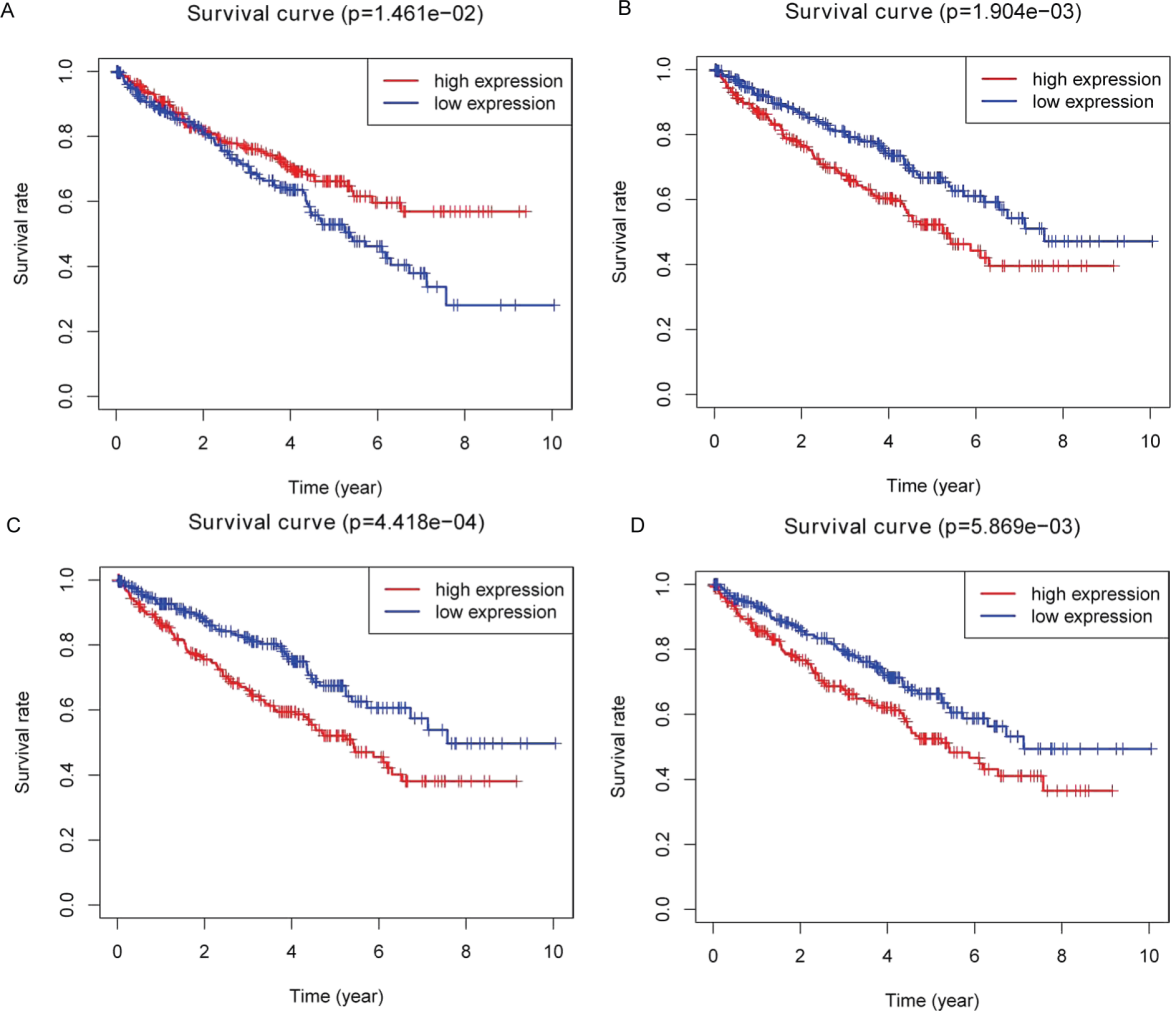
Identification of prognostic key DEMs. We operated overall survival analyses of all 26 key DEMs of ccRCC patieints from TCGA and found four miRNAs that were prognosis-related (miR-21-5p (A), miR-142-3p (B), miR-155-5p (C), miR-342-5p (D)), these prognostic key DEMs were selected for next step.

Kaplan–Meier plots were drawn on the basis of the survival data downloaded from TCGA. Four of 26 key DEMs—miR-21-5p (Fig. 5A), miR-142-3p (Fig. 5B), miR-155-5p (Fig. 5C) and miR-342-5p (Fig. 5D)—were believed to be associated with overall survival (OS) in patients with ccRCC. According to the results, low expression of miR-21-5p and elevated expression of the other three miRNAs in patients with ccRCC might lead to poorer OS.

**Figure 6:**
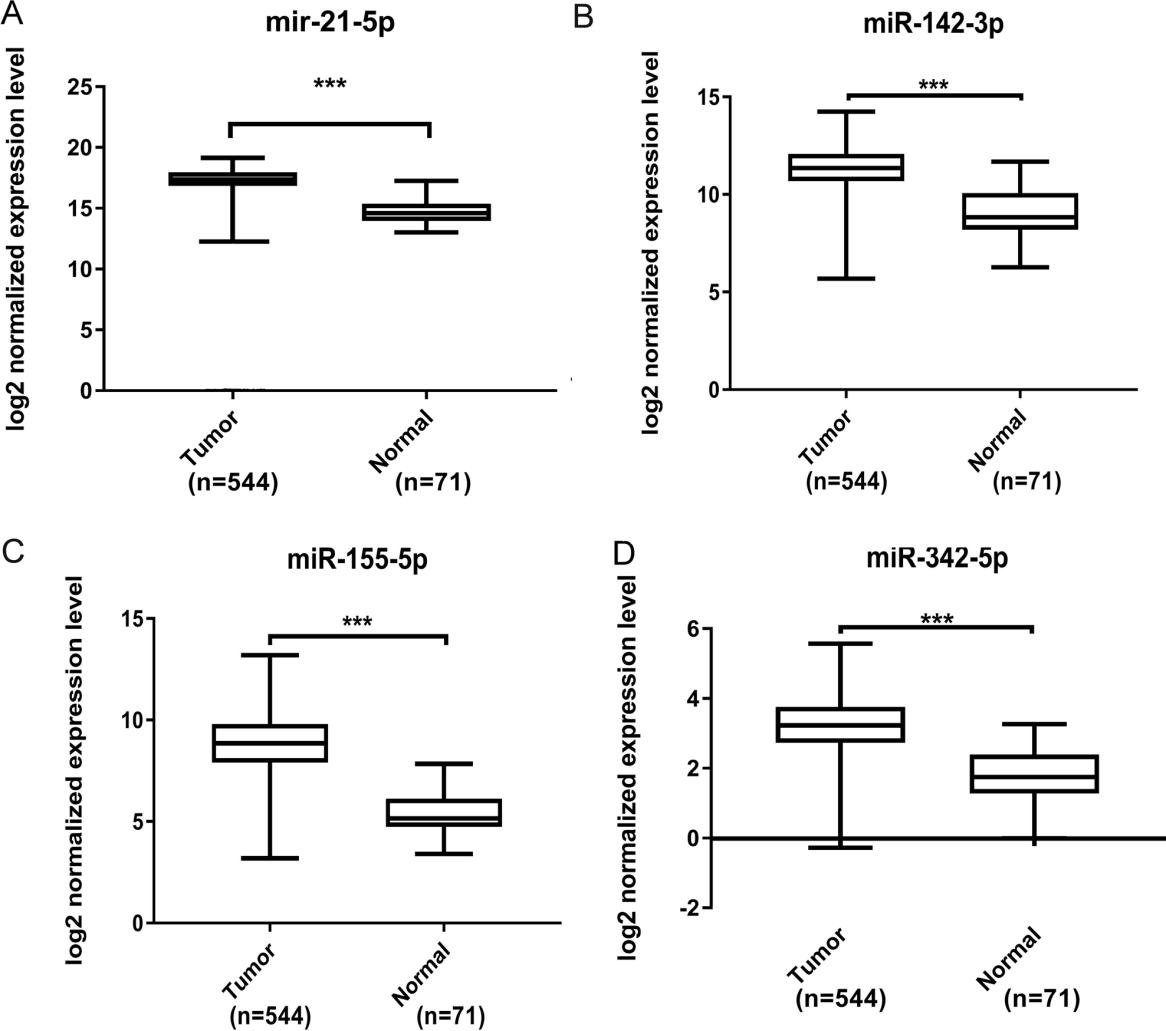
Validation by expression data from TCGA. To validate our results obtained by GEO, we compared the relative expression level of miR-21-5p (A), miR-142-3p (B), miR-155-5p (C), miR-342-5p (D) in ccRCC tissues and normal tissues by expression profiles of TCGA. We identified that all four DEMs showed considerable difference. The *** means *p* < 0.001.

**Figure 7:**
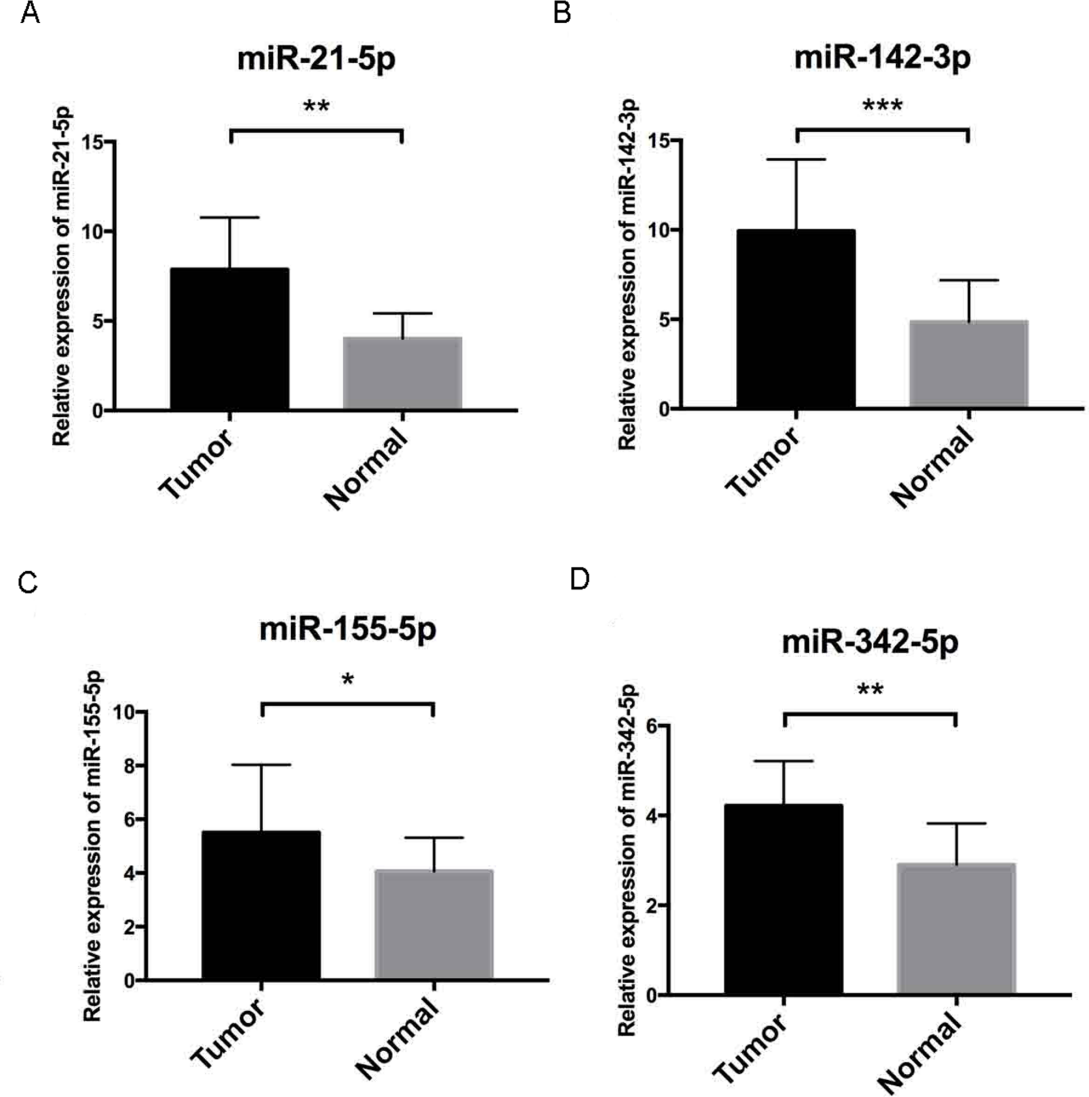
Validation by qRT-PCR. To further validate our results, qRT-PCR of miR-21-5p, miR-142-3p, miR-155-5p and miR-342-5p (Figs. 7A–7D, respectively) were performed, U6 acted as internal control to normalize the results, Cycle threshold (CT) values were calculated after each PCR reaction. Each sample was tested in triplicate and the relative quantification equation (*RQ* = 2 − ΔΔCT) was used for evaluating relative miRNA expression. In this figure, the validation results were concordant with those obtained via bioinformatics methods. In this figure, * is a marker meaning *p* < 0.05, ** means *p* < 0.01, and *** represents *p* < 0.001.

Further validation using TCGA clinical data was then performed with t-tests. The expression levels of all four miRNAs in tumor tissues were notably different from those in normal tissues (*p* < 0.001). The expression levels of miR-21-5p (Fig. 6A), miR-142-3p (Fig. 6B), miR-155-5p (Fig. 6C) and miR-342-5p (Fig. 6D) were dramatically higher in tumor tissues. Box-plots were constructed, and the primary data were log2 standardized to facilitate the visualization of differential expression. Only hsa-miR-21-5p had *p* < 0.05 in both univariate and multivariate analyses (Table S4). We then built a nomogram model (Fig. S1) for better prediction. The areas under the model’s three- and five-year OS receiver operating characteristic curve were 0.759 and 0.714, respectively (Figs. S2A, S2B); the concordance-index (C-index) was 0.717 (95% confidential interval: 0.713–0.721); and the calibration curves (Figs. S3A, S3B) showed high agreement between the predicted and observed OS. Together, these results indicated the accuracy of our nomogram model. The outcomes of qRT-PCR revealed higher expression levels of miR-21-5p, miR-142-3p, miR-155-5p and miR-342-5p in ccRCC specimens (Figs. 7A–7D). These findings were consistent with results based on bioinformatics methods.

## Discussion

A total of 233 ccRCC samples and 193 normal control samples were collected from eight datasets. In aggregate, 26 key DEMs, 167 key upregulated DEGs and 140 key downregulated DEGs were identified from this analysis. Through functional enrichment analyses, associations between key DEGs in ccRCC with common cancer pathways, such as tight junction and HIF-1 signaling pathways, were identified. Because various miRNAs and mRNAs interact with one another, miRNAs were considered to have crucial roles in all identified key cellular pathways.

Among all these key DEMs, miR-21 (Fritz et al., 2014), miR-34a (Fritz et al., 2015), miR-124 (Butz et al., 2015), miR-141 (Liep et al., 2016), miR-146a (Yang et al., 2018), miR-155 (Zhang et al., 2018), miR-200c (Jiang et al., 2016), miR-210 (Samaan et al., 2015), miR-224 (Fujii et al., 2017), miR-452 (Liu et al., 2018) and miR-142 (Liu et al., 2010) have been reported to be correlated with ccRCC. Therefore, our findings are consistent with those from former studies. This consistency, to some extent, justifies our findings. In contrast, other miRNAs, including miR-142, miR-342, miR-363, miR-532 and miR-15a, have not been reported in previous ccRCC studies, although our results suggested their possible connection with ccRCC. Moreover, on the basis of the data for 506 patients with ccRCC in TCGA, the group with high expression of miR-155-5p, miR-142-3p and miR-342-5p showed poorer OS than the low-expression group. In contrast, high miR-21-5p expression was associated with better survival. Therefore, these miRNAs may serve as prognostic factors for patients with ccRCC. The differential expression of these miRNAs was further validated with TCGA database data for 544 ccRCC specimens and 71 normal kidney tissue samples in the “TCGA-KIRC” dataset. Among them, miR-21-5p acts as a post-transcriptional repressor of SATB1 expression and is associated with prognosis in ccRCC (Kowalczyk et al., 2016). Additionally, miR-155-5p has been reported to predict the recurrence and tumorigenesis of kidney renal clear cell carcinoma. Furthermore, its potential oncogenic role in renal cell carcinoma tumorigenesis has been confirmed (Zhang et al., 2018), and increased levels of miR-142-3p can cause loss of function of the tumor suppressor LRRC2 (Liu et al., 2010). In addition, miR-342-5p overexpression may render breast cancer cells less proliferative and more sensitive to cellular stress by affecting HER2 downstream signaling, cell motility and mitochondrial stability (Lindholm et al., 2018). Intensive clinical validation and pathway exploration of these three miRNAs should be performed to discover novel ccRCC mechanisms.

In this study, 167 key upregulated and 140 key downregulated DEGs were filtered on the basis of the interactions of target genes for 26 DEMs and DEGs generated by 5 microarrays. A miRNA-mRNA regulatory network was constructed and provided targets that might potentially be used in evaluating prognosis. miRNA-mRNA integrated analysis has been applied in several studies, which have reported potential targets as prognostic and carcinogenetic biomarkers in prostate cancer (Li, Hao & Song, 2018) and colorectal cancer (Xu et al., 2018). The TCGA validation results indicated that has-miR-21-5p was the only DEM with meaningful outcomes in either univariate or multivariate Cox regression analyses. By adjusting factors in the nomogram model, we found that the inclusion of miR-21-5p in the model increased the area under the curve; therefore, this nomogram model has better prognostic power than prediction based only on stage and grade. This model could be applied in clinical use in the future.

This study has several limitations. First, the miRNA and mRNA data were from various GEO datasets, and the expression in different datasets may have been greatly affected by the detection methods, researcher skill and specimen status. Moreover, among the three miRNA datasets we used, GSE16441 and GSE71302 are based on the same microarray, whereas GSE116251 is based on another microarray. Therefore, technical and chemistry specific differences might have outweighed differences in biology in the process of DEM selection. Most samples in this study were from American patients, which may have led to a risk of selection bias. Additionally, future in vitro and vivo experiments are needed for validation.

## Conclusion

In conclusion, we identified 26 miRNAs that may participate in key pathways including tight junction and HIF-1 signaling pathways in ccRCC regulatory networks. We found that higher expression of miR-142-3p, miR-155-5p and miR-342-5p, and lower expression of miR-21-5p were associated with poor survival in the prognosis of ccRCC. Additionally, a nomogram model composed of independent prognostic factors (has-miR-21-5p, stage and grade) showed strong prognosis prediction ability. Our results indicate a need for further experimental studies for validation.

##  Supplemental Information

10.7717/peerj.10292/supp-1 Supplemental Information 1 26 key DEMs and expression data from the extracted datasets The list of 26 key DEMs and the expression data from the extracted datasets ( GSE71302, GSE16441, GSE116251). The normal samples were highlighted in yellow to distinguish from the tumor samples, and the expression profiles from GSE116251 were log2 normalized in accordance with data from the other two datasets.

10.7717/peerj.10292/supp-2 Table S1 General characteristics of the patients involved in PCR validation This table presented the clinical and pathologic characteristics of the patients characterized using qRT-PCR.

10.7717/peerj.10292/supp-3 Table S2 Realtime PCR primers The primers sequences of all four miRNAs that presented significant results in overall survival analysis and the primer sequences of U6 (which was used as an internal control to normalize the results).

10.7717/peerj.10292/supp-4 Table S3 Expression profiles of four key DEMs In this table, each sheet represents the detection of expressions of the five key microRNAs in 15 pairs of ccRCC samples and matched adjacent normal kidney tissues ( *n* = 30) from patients who received radical nephrectomy from January 2017 to March 2018 in Shengjing Hospital of China Medical University. All sheets were labeled by their corresponding miRNAs respectively.

10.7717/peerj.10292/supp-5 Table S4 Univariate and multivariate cox analyses of hsa-miR-21-5p Based on the results of univariate and multivariate cox regression analyses of clinicopathological characteristics (age, stage, grade, etc.) and four prognostic key DEMs which were screened by overall survival analysis, we found that only miR-21-5p presented significant results in both univariate and multivariate analyses, in this table, sheet 1 and sheet 2 showed the results of univariate and multivariate cox analyses respectively.

10.7717/peerj.10292/supp-6 Figure S1 A nomogram model based on miR-21-5p We created a nomogram model based on the expression of hsa-miR-21-5p, grade and stage for better predicability of 3 and 5-year overall survival of ccRCC patients.

10.7717/peerj.10292/supp-7 Figure S2 ROC curves of nomogram models The 3 and 5-year overall survival ROC curves of nomogram model based on hsa-miR-21-5p, stage and grade (A, B) and those of nomogram model consists of only grade and stage (C, D). The AUC values of the model containing hsa-miR-21-5p were higher than the results of the model where it was excluded, which justified that the inclusion of hsa-miR-21-5p could increase the prognostic predictability of the model.

10.7717/peerj.10292/supp-8 Figure S3 Calibration curves of the nomogram model Apart from the ROC curves in Fig. S2, we also depicted 3 and 5-year calibration curves to further validate the predictability of the model. According to the calibration curves, we could tell that the predicted and observed overall survival were of great accordance.

## Additional Information and Declarations

### Field Study Permissions

The following information was supplied relating to field study approvals (i.e., approving body and any reference numbers): This study was approved by Shengjing Hospital Ethics Committee (2017PS012J).

### Data Availability

The following information was supplied regarding data availability: Data is available at TCGA (TCGA-KIRC) and GEO (GSE16441–GPL8659, GSE71302, GSE116251, GSE15641, GSE16441–GPL6480, GSE16449, GSE53757, GSE68417). Raw data of qRT-PCR validation are available as a Supplemental File.
