# Supplementary material for: Identification of the miRNA-mRNA regulatory pathways and a miR-21-5p based nomogram model in clear cell renal cell carcinoma
Source: PeerJ. 2020 Nov 4;8:e10292. doi: 10.7717/peerj.10292 (PMC7648458; doi:10.7717/peerj.10292)
Supplement: Table S1 — This table presented the clinical and pathologic characteristics of the patients characterized using qRT-PCR. [file peerj-08-10292-s002.docx]

General characteristics of the patients involved in PCR validation

| Characteristics | N（%） |
| --- | --- |
| Age （years） |  |
| <60 | 6 (40) |
| ≥60 | 9 (60) |
| Gender |  |
| Male | 11 (73.3) |
| Female | 4 (26.7) |
| ECOG score |  |
| ≤1 | 12 (80) |
| ≥2 | 3 (20) |
| T stage |  |
| T1 | 0 (0) |
| T2 | 9 (60) |
| T3 | 5 (33.3) |
| T4 | 1 (6.7) |
| N stage |  |
| N0 | 14 (93.3) |
| N1 | 1 (6.7) |
| M stage |  |
| M0 | 15 (100) |
| M1 | 0 (0) |
| Histological grade |  |
| G1 | 0(0) |
| G2 | 10 (66.7) |
| G3 | 4 (26.7) |
| G4 | 1 (6.7) |
