# Supplementary material for: Identification of the miRNA-mRNA regulatory pathways and a miR-21-5p based nomogram model in clear cell renal cell carcinoma
Source: PeerJ. 2020 Nov 4;8:e10292. doi: 10.7717/peerj.10292 (PMC7648458; doi:10.7717/peerj.10292)
Supplement: Table S2 — The primers sequences of all four miRNAs that presented significant results in overall survival analysis and the primer sequences of U6 (which was used as an internal control to normalize the results). [file peerj-08-10292-s003.docx]

Realtime PCR primers

|  | primer sequences (5'-3') |
| --- | --- |
| miR-21-5p | Forward: GCCCGCTAGCTTATCAGACTGATG  Reverse: GTGCAGGGTCCGAGGT |
| miR-142-3p | Forward: UGUAGUGUUUCCUACUUUAUGGA  Reverse: CAUAAAGUAGGAAACACUACAUU |
| miR-155-5p | Forward: GGGTTAATGCTAATCGTGATA  Reverse: CAGTGCGTGTCGTGGAGT |
| miR-342-5p | Forward: CGGAGGGGTGCTATCTGTGATTGAG  Reverse: CAATGGATCCGACATAGTC  Reverse: |
| U6 | Forward: CTCGCTTCGGCAGCACA Reverse: AACGCTTCACGAATTTGCGT |
