# Supplementary figures and images for: Identification of the miRNA-mRNA regulatory pathways and a miR-21-5p based nomogram model in clear cell renal cell carcinoma
Source: PeerJ. 2020 Nov 4;8:e10292. doi: 10.7717/peerj.10292 (PMC7648458; doi:10.7717/peerj.10292)

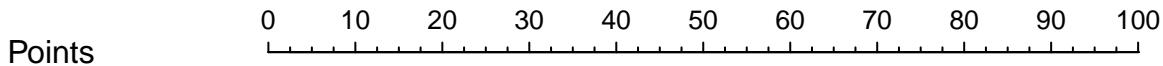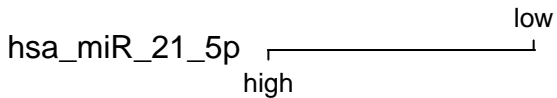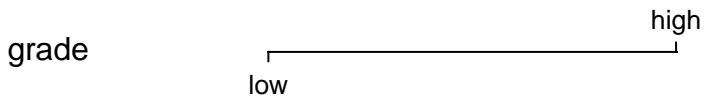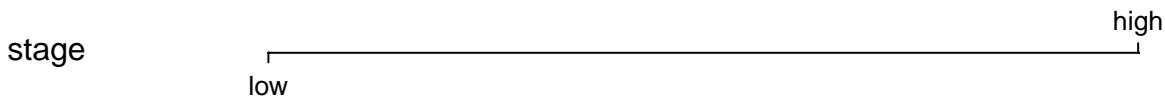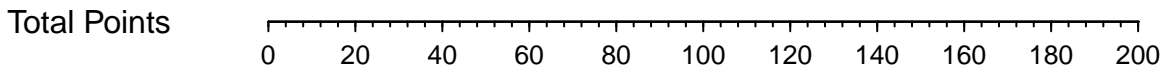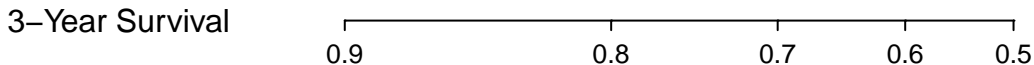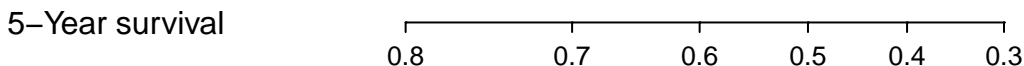

Supplement: Figure S1 — We created a nomogram model based on the expression of hsa-miR-21-5p, grade and stage for better predicability of 3 and 5-year overall survival of ccRCC patients. [file peerj-08-10292-s006.pdf]

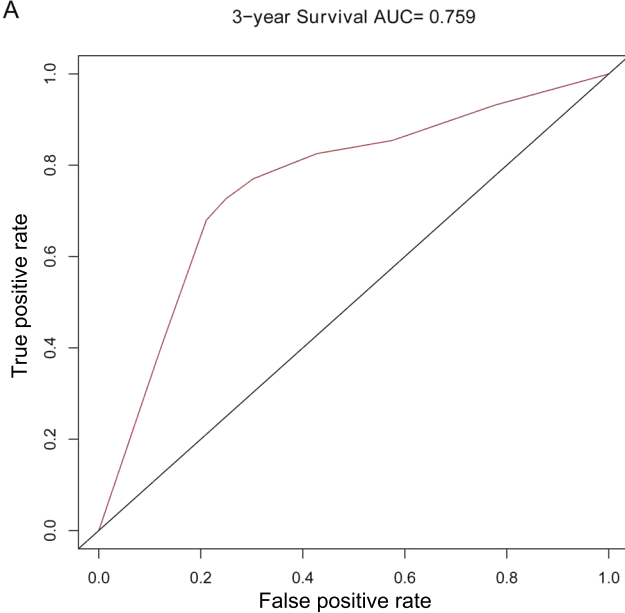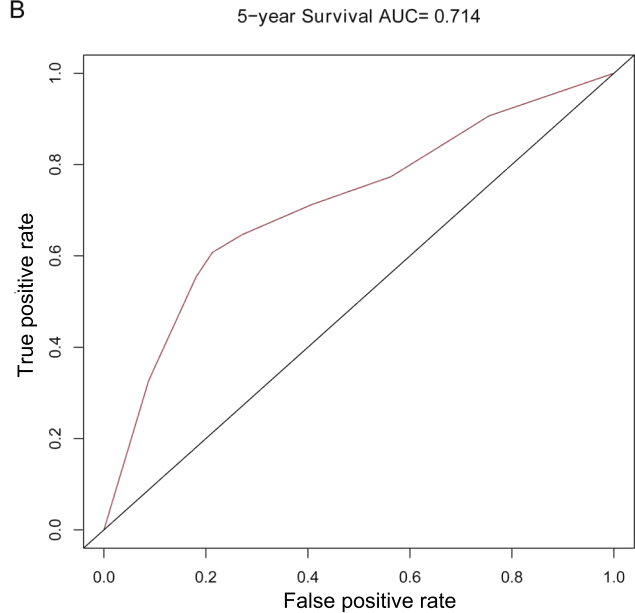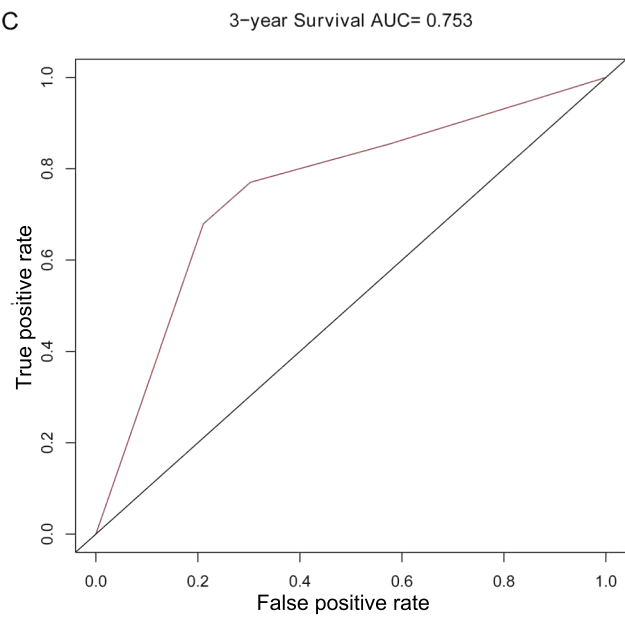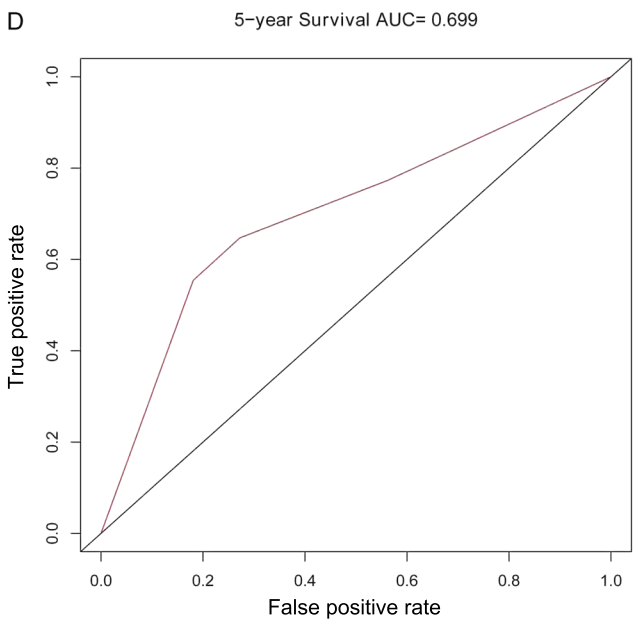

Supplement: Figure S2 — The 3 and 5-year overall survival ROC curves of nomogram model based on hsa-miR-21-5p, stage and grade (A, B) and those of nomogram model consists of only grade and stage (C, D). The AUC values of the model containing hsa-miR-21-5p were higher than the results of the model where it was excluded, which justified that the inclusion of hsa-miR-21-5p could increase the prognostic predictability of the model. [file peerj-08-10292-s007.pdf]

A

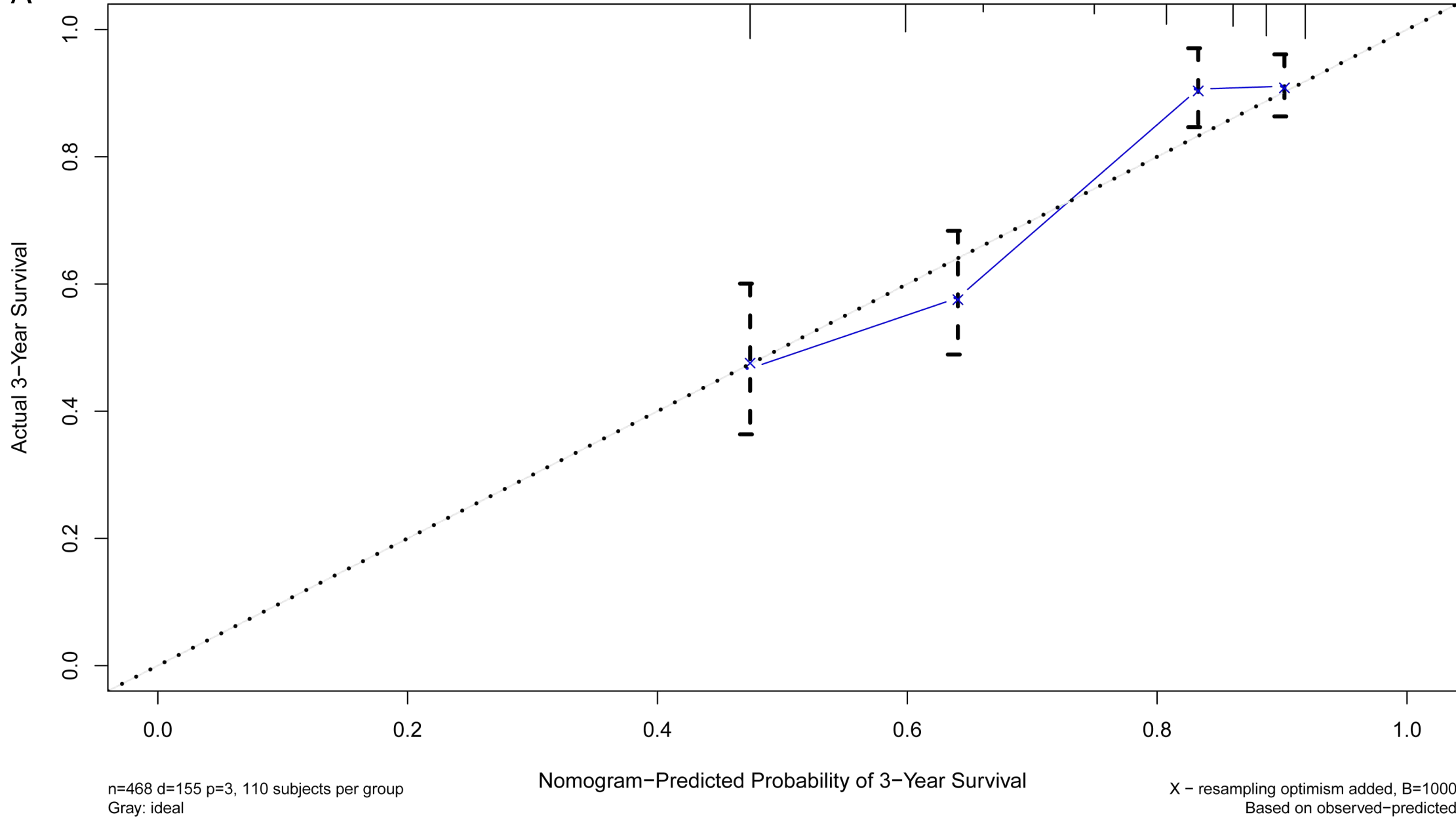

B

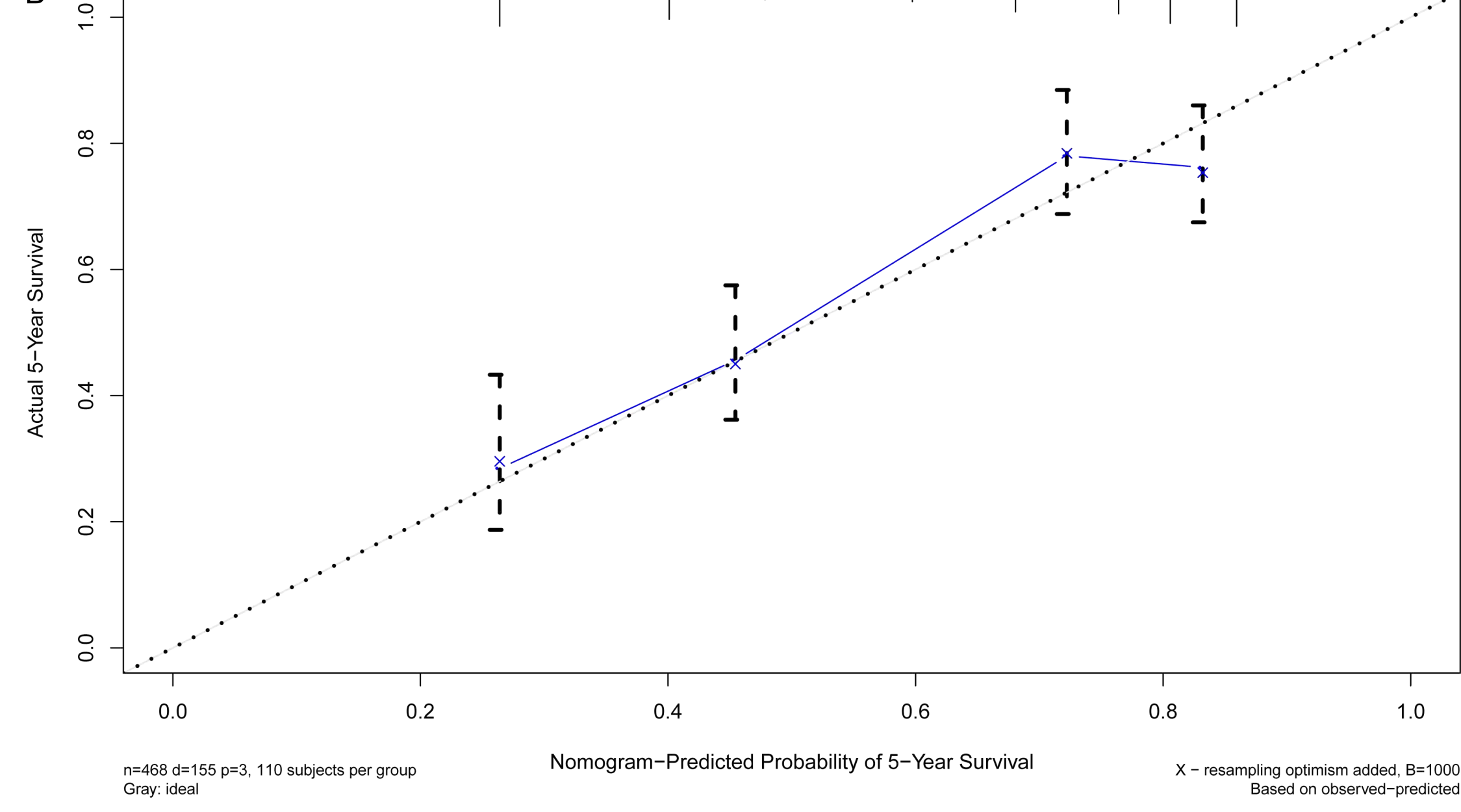

Supplement: Figure S3 — Apart from the ROC curves in Fig. S2, we also depicted 3 and 5-year calibration curves to further validate the predictability of the model. According to the calibration curves, we could tell that the predicted and observed overall survival were of great accordance. [file peerj-08-10292-s008.pdf]
